# Credible knowledge: A pilot evaluation of a modified GRADE method using parent-implemented interventions for children with autism

**DOI:** 10.1186/1472-6963-11-60

**Published:** 2011-03-22

**Authors:** J Michael Van Adel, Jennifer Dunn Geier, Adrienne Perry, Jo-Ann M Reitzel

**Affiliations:** 1 The Provincial Centre of Excellence for Child and Youth Mental Health at CHEO, Ottawa, ON, Canada; 2 Autism Intervention Program - Eastern Ontario, Children's Hospital of Eastern Ontario, Ottawa, ON, Canada; 3 Department of Psychology, York University, Downsview, ON, Canada; 4 McMaster Children's Hospital, Hamilton-Niagara Regional Autism Intervention Program, Hamilton, ON, Canada

## Abstract

**Background:**

Decision-making in child and youth mental health (CYMH) care requires recommendations that are developed through an efficient and effective method and are based on credible knowledge. Credible knowledge is informed by two sources: scientific evidence, and practice-based evidence, that reflects the "real world" experience of service providers. Current approaches to developing these recommendations in relation to CYMH will typically include evidence from one source or the other but do not have an objective method to combine the two. To this end, a modified version of the Grading Recommendations Assessment, Development and Evaluation (GRADE) approach was pilot-tested, a novel method for the CYMH field.

**Methods:**

GRADE has an explicit methodology that relies on input from scientific evidence as well as a panel of experts. The panel established the quality of evidence and derived detailed recommendations regarding the organization and delivery of mental health care for children and youth or their caregivers. In this study a modified GRADE method was used to provide precise recommendations based on a specific CYMH question (i.e. What is the current credible knowledge concerning the effects of parent-implemented, early intervention with their autistic children?).

**Results:**

Overall, it appeared that early, parent-implemented interventions for autism result in positive effects that outweigh any undesirable effects. However, as opposed to overall recommendations, the heterogeneity of the evidence required that recommendations be specific to particular interventions, based on the questions of whether the benefits of a particular intervention outweighs its harms.

**Conclusions:**

This pilot project provided evidence that a modified GRADE method may be an effective and practical approach to making recommendations in CYMH, based on credible knowledge. Key strengths of the process included separating the assessments of the quality of the evidence and the strength of recommendations, transparency in decision-making, and the objectivity of the methods. Most importantly, this method combined the evidence and clinical experience in a more timely, explicit and simple process as compared to previous approaches. The strengths, limitations and modifications of the approach as they pertain to CYMH, are discussed.

## Background

An essential ingredient in effective clinical practice is that clinicians benefit, in a timely way, from current research knowledge in their fields. Those responsible for the organization and delivery of child and youth mental health (CYMH) care require such knowledge, which is rapidly changing and ever increasing. Moreover, clinicians benefit from knowledge that is credible. This is especially critical in the development of clinical recommendations, which are intended to achieve the linked objectives of improvements in patient or client outcome and the efficient use of resources. To be credible, these recommendations must consider input from two sources: scientific evidence and the practice-based evidence, reflecting the "real world" expertise and experience of service providers.

Clearly, it is less than practical to expect that individual clinicians can continuously survey and evaluate available knowledge and adapt clinical decisions accordingly. Despite this recognition, however, there are still obstacles that need to be overcome in improving the access to and practical application of credible knowledge in CYMH. Primarily, there is a lack of agreement about the best methods for organizations to identify, assess, and synthesize relevant studies [1-7]. Without a useful methodology for identification and evaluation, published research results may not be adequately assessed for their scientific validity. Poor research, which can suffer from serious methodological flaws, may then be relied upon to make critical treatment decisions. On the other hand, when recommendations are based on rigorous evaluation of only high quality evidence, this evidence may not be assessed for its relevance and practical application to specific care and may not be suitable for generalization to clinicians' practice. These flaws are present in the current approaches to synthesizing evidence-based practice in CYMH.

Numerous professional bodies and scientific organizations have taken the approach of developing clinical practice guidelines. These guidelines include recommendations to aid patient and practitioner decision-making in specific clinical situations. A strength of this approach is that a panel of experts typically develops these recommendations. Panel members have access to the evidence, an understanding of the clinical problems and research methods, and sufficient time for deliberation. However, the recommendations are often formulated without systematically identifying and summarizing the evidence [8]. A proper evaluation of the evidence is critical in ensuring the validity of guidelines. Otherwise, relevant studies may be missed and recommendations made which contradict the current best available evidence. Furthermore, when more rigorous and transparent methods are used, guideline development can take two years or more. As a result, these guidelines can require considerable financial and human resources and the timeline may result in a delay in getting pertinent information to health care providers, thereby having a potential impact on clinical practice [4,9,10].

Systematic reviews take a different approach by using explicit search strategies and inclusion criteria to identify and judge evidence of the effectiveness of clinical interventions [11]. The relative comprehensiveness of these reviews is a key strength. Null or even negative findings are often included, whereas in other approaches such research has been largely ignored in the accumulation of supportive findings [12]. However, health professionals, physicians, and policy makers infrequently refer to these reviews in decision-making [13-15]. So, despite the increasing summaries of the evidence in systematic reviews and clinical practice guidelines, as many as 30 to 45 per cent of patients fail to receive evidence-based treatment, leading to less than optimal outcomes [16-18]. This is due to a number of limitations in these reviews [11]; perhaps the most significant one being the lack of a well-defined, transparent process for including the input of relevant evidence users that have practice-based knowledge. This limitation results in the omission of input from those intended to implement and benefit from the recommendations.

Input from practicing health professionals can make recommendations more practical and clinically- relevant. This can be accomplished through an approach that integrates scientific evidence with the knowledge of patients' values and preferences as well as the implications for treatment planning and decision-making in clinical practice.

### A New Approach

The Grading of Recommendations, Assessment, Development, and Evaluation (GRADE) approach [19] incorporates the strengths of previous approaches and accounts for their limitations. It may be the more effective method for providing credible knowledge that is essential to CYMH.

GRADE employs a sequential assessment of 1) the quality of the evidence, followed by 2) an evaluation of the balance between benefits and harms and 3) a subsequent judgment about the strength of recommendations [20]. The separation of judgments on the quality of evidence and the strength of recommendations is a critical and defining feature of this method.

GRADE was developed based on a systematic review that identified the fundamental elements of an effective evidence grading system. These elements include: the rating of the quality of individual studies; the quantity in terms of magnitude of effect, number of studies and sample size; and the consistency of the findings between similar and different study designs [21].

The current approaches to synthesizing evidence-based practice research in the CYMH field should be more appropriately described as clinical treatment efficacy research [22]. In the past, these approaches have relied on a one-to-one fit between the research on effective practices and the implementation of these practices in routine care. One clear solution to this problem would see research conducted that attends to service delivery issues at the outset. However, until that sort of evidence becomes available existing evidence-based practice research would be left collecting dust on academic shelves. Instead, the GRADE method may provide a method that can make use of the wealth of existing research. The GRADE approach attempts to merge research-based knowledge of efficacy with practice-based knowledge of effectiveness through the input of the expert panel.

The process begins with the selection of a focused question that can lead to action and the establishment of an expert panel with knowledge related to the question. Ideally, and for the sake of efficiency, relevant published and unpublished scientific evidence relating to the question is gathered from an existing systematic review. A list of potentially important outcomes is then created which panel members must independently rate according to importance ranging from unimportant to critical. The improvement of outcomes with high patient importance (i.e., considered critical) should lead to stronger recommendations [23].

Following the rating of the importance of outcomes, the quality of the evidence is then rated. A number of factors can increase or decrease the quality rating and this should reflect the confidence in the studies' estimates of benefits and harms.

Finally, the evidence, its quality rating and the outcome information is synthesized succinctly and transparently in evidence profiles. These profiles guide the panel in deciding on the strength of recommendation according to the tradeoffs between benefits and harms of the various alternatives.

Based on conceptual arguments and past experience with the process, many developers and users of GRADE believe that this process can be applied across all forms of intervention [24]. Others have argued that a single system cannot adequately address evidence across a wide range of contexts without being too complex to be useful [25,26]. Until now, GRADE has been used primarily to address topics in the field of medicine [e.g., [23,27,28]]. CYMH is an area in need of a more efficient and effective approach to producing credible knowledge. Furthermore, it is an area in which the potential of the GRADE method has not been evaluated.

### Current Study

Against this backdrop, the Provincial Centre of Excellence for Child and Youth Mental Health at the Children's Hospital of Eastern Ontario completed a pilot project to investigate the use of a streamlined version of the GRADE approach. Autism intervention research was identified as a suitable and interesting area in which to pilot test this methodology. Specifically, this streamlined method was used to determine current credible knowledge concerning the effects of parent-implemented, early intervention for children with autism. Implementing a methodology such as GRADE has considerable implications for improving efficiency, communication, and clinical decision-making. GRADE may facilitate the progression of the CYMH field toward greater use of evidence-based practice by service providers. This pilot study is the first step in evaluating the feasibility of this methodology as applied to CYMH research and determining whether any modifications are necessary in its application to this field. The findings related to autism as well as the strengths and limitations of the approach will be discussed.

### Autism Research

The history of controversy surrounding autism makes this area suitable for this pilot project and is certainly illustrative of the importance of an established methodology for determining credible knowledge in the field of CYMH. Autism research has, for example, seen the persistence of a dispute surrounding evidence concerning causal linkages associated with the measles-mumps-rubella (MMR) vaccine or the vaccine preservative thimerosal. Notwithstanding, there has been no evidence to support an association between an MMR vaccine and autism, nor is there a plausible biological mechanism [as reviewed by 29]. Valuable time and resources were used to conduct as many as twenty studies to definitively respond to a study [30], whose arguments had many flaws that need not be explored here [29,31,32].

There has also been intense debate surrounding the evidence base and suitability of the variety of educational strategies and interventions that have been developed for individuals with autism [33-37]. For instance, controversy exists regarding the necessary intensity of intervention to achieve positive outcomes, the use of certain outcomes and the efficacy of one program compared to another [38]. Some approaches have also been popular with parents, but many professionals have not embraced them enthusiastically, again leading to considerable controversy [39]. While debate is clearly healthy in furthering the scientific research in relation to these practices, the level of uncertainty in this area has resulted in professionals, parents, and policy makers receiving confusing and sometimes unreliable information about the relative effectiveness of interventions [6].

Narrative reviews of interventions in this area have sought to address this problem by identifying evidence-based treatments. Unfortunately, many of these reviews have not taken a systematic approach, limiting their comprehensiveness and validity [33,35,40-44]. Alternatively, systematic reviews have included a wider range of evidence but most have also been found to suffer from methodological weaknesses that make them susceptible to bias [45]. Other reports have taken a systematic approach to the scientific evidence along with expert clinical opinion and took many years to complete. For instance, the National Autism Centre [46] recently completed a review of effective treatments for autism. This review took four years and required substantial resources. Still, others using this approach could not provide precise recommendations regarding specific interventions.

The National Research Council [36] and New York State Department of Health [47] each conducted a systematic review and developed clinical practice guidelines intended to identify effective practices. These reports did not recommend specific interventions. The reports cited a lack of well-controlled studies and an inability to compare interventions between studies. This was stated to be a result of varying descriptions of interventions, participants, treatment fidelity and other methodological considerations. Instead, both reports identified several key features of successful approaches to the education of children with autism, including early intervention after diagnosis, intensive instructional programming, as well as parental involvement in educating their child.

Parent-implemented interventions have been a particular focus in recent reviews, as increased parental skills allow for continual opportunities for children's learning across a range of situations [48,49]. Even in using a more narrowly defined scope of interventions for children with autism, the authors of these reviews were unable to provide recommendations as to specific interventions and offered few implications for practice. This study aims to show that a modified GRADE approach can improve on these attempts at developing recommendations when applied to a CYMH topic.

## Methods

### The Grade Approach

The process used for this pilot project was adapted from that described by the GRADE working group and is more fully described elsewhere [19,24,50-53]. The approach represents an explicit assessment of the quality of evidence, the balance between benefits and risks and the strength of recommendations. Separating the judgments of the quality of the evidence and the strength of recommendations is a critical and defining aspect of GRADE.

Adjustments to the process were made throughout the course of the pilot project to adapt the method in answering a CYMH question. Any changes to the process are noted. The question selected for this pilot project was: *What is the current credible knowledge concerning the effects of parent-implemented, early intervention for their children with autism?*

A detailed literature search was performed for existing systematic reviews that addressed this question. Unfortunately, systematic reviews by Diggle, McConachie & Randle [48] and McConachie & Diggle [49] related to this question used eligibility criteria that may have been too narrow. A limited number of heterogeneous studies were identified that were not comparable, and did not lead to specific and precise recommendations [54]. Specifically, the former excluded studies that used non-random allocation of groups, while both studied children aged only one to six years with any diagnosis on the autism spectrum. Broader criteria could lead to more informative recommendations by including non-randomized studies and a wider age range, resulting in a broader range of interventions reviewed. As a result, eligibility criteria were specified independent of the systematic reviews, as follows:

(1) Families had to have at least one child who received a formal diagnosis of autism.

(2) Parent-implemented: At least one parent was formally trained to deliver an intervention for their child with autism, which was different in some way from any intervention(s) delivered by service providers.

(3) Early intervention: At the time of study initiation, within each study group at least one recipient of a parent-implemented intervention had been under eight years of age.

(4) Research designs: prospective and controlled.

(5) All publication dates considered.

(6) Effects: any outcomes (e.g., child, interaction, parent, system-related), which includes benefits and harms.

Based on these criteria, an independent information specialist searched the following databases: ERIC, The Cochrane Library, MEDLINE, EMBASE, PsycInfo, CINAHL, and Dissertation Abstracts International. Other sources of information were examined including the bibliographies of systematic and narrative reviews and reference lists of key articles identified through the search strategy. Screening by three independent screeners of the results yielded 11 primary studies to be included [55-65]. 	This deviates from the original GRADE method in that the evidence-base is typically independently established by an existing systematic review in a GRADE review. Given the issues with the existing systematic reviews on this topic (noted above), the procedure was modified in the current study to have an independent information specialist search for individual studies. This subsequently modifies the procedure for appraising the evidence, as described below.

A panel, which included three clinical and methodological experts in autism research, was then formed. In this pilot project, one role of the panel was to serve as a proxy for end users in determining the values and preferences of those end users in rating the importance of outcomes. The inclusion of additional members was beyond the resources of this work.

### Outcome Ratings

In addition to using the panel's experience, an extensive literature search was performed to identify preference estimates from population-based studies in order to gain additional, evidence-based, perspectives in rating the importance of outcomes. These studies are intended to provide information that reflects the values, beliefs and preferences of those affected by autism. The panel could use these studies in rating the importance of outcomes. The panel then created a list of potentially important outcomes to rate, independent of the primary studies. Outcomes are rated on a scale from 1-9: a rating of 7-9 indicating that the outcome is critical for a decision or recommendation; a rating of 4-6 indicating it is important; and, a rating of 1-3 indicating it is not important.

### Quality of Evidence

Quality of the evidence was then assessed using the GRADE four-category system (high, moderate, low and very low quality). The quality of the evidence reflects the extent to which one can be confident that the estimates of the benefits and harms of a researched intervention are accurate. Evidence based on randomized controlled trials begins with a high quality rating, observational studies with a low rating and any other evidence with a very low rating. However, studies may then be upgraded or downgraded. Five limitations--related to study quality, consistency, directness, precision, and reporting bias--may lead to downgrading. Upgrading the quality of the evidence may result from large effects, dose-response gradient and if all plausible confounding variables or biases would decrease the demonstrated effect, such as only sicker patients receiving an experimental intervention, yet still faring better. It is important to note that evidence that is downgraded for any reason cannot be upgraded. To ensure transparency and objectivity, the panel provided explicit reasons for downgrading and upgrading by including footnotes in the evidence profiles; this is a requirement of the GRADE system.

Due to the heterogeneity of the evidence base for the present application, namely that each study evaluated a different intervention using a different set of outcomes along with variations in the specific populations studied and those implementing the interventions, study results could not be combined nor directly compared. This diverged from the typical GRADE method where studies evaluating the same outcomes are combined and the quality of each outcome is then evaluated separately. In the present modified version of GRADE, studies were individually appraised for quality on a number of dimensions (see Table 1). The labels high, moderate, low and very low quality evidence were then applied to individual studies as opposed to a synthesized body of evidence, as in the original GRADE method. For each intervention considered, the panel then formulated a consensus recommendation based on the panel members' judgments regarding the balance between the benefits, harms, and values and preferences (i.e., the desirability or preference that individuals exhibit for a particular outcome) of the intervention. Due to the limited resources of this pilot-project, information on costs (i.e., resource utilization) were not acquired, and they were not considered when making a recommendation.

**Table 1:** Factors assessed in critically appraising the evidence

Factors that might decrease quality of evidence	Factors that might increase quality of evidence
• Lack of allocation concealment • Lack of blinding (particularly for subjective outcomes) • Failure to adhere to an intention to treat analysis • Stopping early for benefit • Selective outcome reporting • Poor matching of groups in nonrandomized studies or inappropriate comparison group (e.g., children with more serious behavioural problems placed in control group) • Absence of treatment fidelity assessment • Inadequate description of intervention and those delivering intervention • Insufficient follow-up period or lack of reporting on losses to follow-up	• Large magnitude of effect • Plausible confounding, which would reduce a demonstrated effect • Dose-response gradient (increased intensity of treatment leads to enhanced benefits)

### Strength of Recommendations

The strength of recommendations was then determined according to the tradeoffs between benefits and harms of the various alternatives. The strength of a recommendation reflects the extent to which one can be confident that the desirable effects of an intervention outweigh the undesirable effects. Although the level of confidence is a continuum, the GRADE system offers two levels of recommendations for or against treatments (i.e., strong and weak), according to the extent of difference between benefits and harms. The strength of a recommendation is also influenced by the values and preferences of end users, in that critical outcomes are the primary factors for establishing a recommendation. This allows the GRADE system to emphasize that weak recommendations in the face of high quality evidence and vice versa, can occur. This is because other factors, beyond the quality of the evidence, including the values and preferences of the relevant population and the importance of the outcomes, impact the strength of a recommendation [20]. Given that the heterogeneity of the evidence did not allow for a synthesis of the evidence, the labels strong and weak were applied to individual interventions, as opposed to the body of the evidence as in the original GRADE method.

Recommendations included details about the population and intervention. This should aid in the interpretation of the strength of the recommendations and help clinical decision-makers deal with individual patients. Given that some of the judgments leading to the recommendations are subjective, the decisions made are explicit and transparent in the remarks that go along with the recommendations. Specific suggestions for new studies to be undertaken were also noted (see example in Additional file 1).

## Results

It took approximately four months to complete this pilot project. A clear answer to the original question as to which parent-implemented intervention is the most effective, was not reached due to the heterogeneity of the studies included (see Table 2 for a description of the interventions). The content of the interventions evaluated differed in almost all studies, while different sets of outcome measures were also used. Any combination or meta-analysis of the data was simply not possible. As a result, specific recommendations were made for each intervention studied. These detailed recommendations included a summary of each piece of evidence with emphasis on the key characteristics (e.g., age group studied, outcomes showing improvement, etc.) influencing a recommendation identified by the panel (see example in Additional file 1).

**Table 2:** Descriptions of the studied interventions

Study	Intervention	Sample Size
Aldred et al. (2005)	Therapist delivered, manual-based program for training and educating parents in adapted communication.	Treatment = 14 Control = 14

Drew et al. (2003)	Speech and language therapist delivered program aimed to train parents to develop their child's joint attention skills and joint action routines.	Treatment = 12 Control = 12

Howlin & Rutter (1987)	A behavioural, linguistic and social training program used to teach parents language modification techniques, management training and behavioural procedures.	Treatment = 16 Control = 16

Koegel et al. (1996)	Pivotal Response Training, a naturalistic paradigm emphasizing motivational procedures and responsivity to multiple cues to improve parent-child interaction.	Treatment = 7 Control = 10

McConachie et al. (2005)	"More Than Words" course aimed at improving parents' understanding of autism and facilitating communication.	Treatment = 26 Control = 25

Neef (1997)	A pyramidal model of parent training by peers to instruct parents in making use of skill training opportunities during daily routines.	Treatment = 14 Control = 12

Ozonoff & Cathcart (1998)	Individualized TEACCH-based home program focusing on cognitive, academic, and prevocational skills.	Treatment = 11 Control = 11

Rickards et al. (2000)	Specialist pre-school teacher delivered, home-based program using discussion, direct skills training and adaptation of the home environment to the child.	Treatment = 18 Control = 21

Schreibman et al. (1995)	Pivotal Response Training, a naturalistic paradigm emphasizing motivational procedures and responsivity to multiple cues to improve parent-child interaction.	Treatment = 10 Control = 7

Tonge et al. (2001)	Special educator or psychologist delivered, manual-based, parent education and behaviour management intervention.	Treatment = 35 Control = 35

Wang (2008)	The author delivered a program aimed at improving the interactive skills of parents using applied behaviour analysis and naturalistic teaching principles	Treatment = 14 Control = 11

**Figure 1:**
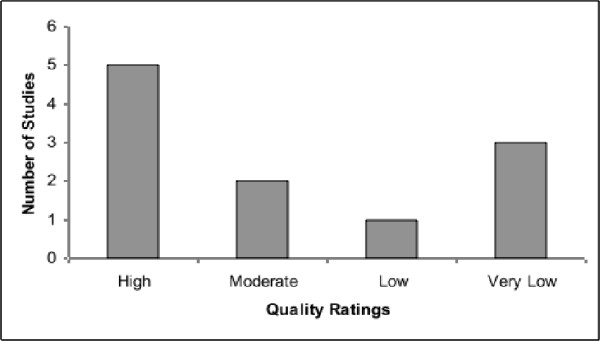
**Quality Ratings. **Frequency count of the quality of the evidence as rated by the panel

Overall, the quality of the evidence ranged from high to very low, based on seven randomized controlled trials and four observational, or quasi-experimental, studies (see Figure 1). The factors that led to downgrading the quality of some studies included a lack of blinding, inappropriate matching of control groups and/or lack of clarity in describing the population, intervention and/or study design. A "weak positive" recommendation was made for each of the eight interventions compared to a no intervention or local services control group. A "weak positive" recommendation was also made for one intervention in each of the four studies comparing interventions; one study compared an intervention to both a group receiving local services and a group receiving a different intervention. All of the studies provided some evidence in favour of the studied intervention, as each demonstrated some positive effects and the remaining showing no significant change. Notably, no study found evidence of any harmful effects of the interventions (see Table 3 for a summary of the evidence and recommendations). Given that costs were not considered and each study demonstrated positive effects and no harms, our results suggest that parent-implemented interventions for children with autism are likely to be beneficial. The exclusion of costs is very likely to have had an effect on the strength of recommendations, as studies were of varying quality, though evidence of benefits on an outcome were required in order to recommend an intervention. By completing this process, gaps and weaknesses in this specific area of research were also identified.

**Table 3:** Summary of the Evidence

Study	Quality Rating	Factors affecting quality Strengths (+)/Weaknesses(-)	Recommendation*	Best Estimates
Aldred et al. (2005)	High	+ RCT + Independent randomization + Allocation Concealment/blinding + Clear outcome reporting + Low risk of bias + Valid outcome measures	Weak +	Improved symptom severity, social communication skills and parent perception of child's communication skills

Drew et al. (2002)	Moderate	+ RCT + Appropriate randomization + Clear outcome reporting + Low risk of bias +Valid outcome measures - poorly matched groups (gender) - lack of blinding	Weak +	Improved Communication Skills

Howlin & Rutter (1987)	Low	+ Appropriately matched control group + Allocation Concealment/blinding + Clear outcome reporting + Low risk of bias + Valid outcome measures - Quasi-Experimental design	Weak +	Improved mother/child speech and interaction

Koegel et al. (1996)	High	+ RCT + Appropriate randomization + Allocation Concealment/blinding + Clear outcome reporting + Low risk of bias + Valid outcome measures	Weak +	Improved parents' global style of interaction with their child

McConachie et al. (2005)	Low	+ RCT + Appropriately matched control group + Allocation Concealment/blinding + Clear outcome reporting + Low risk of bias + Valid outcome measures - Quasi-Experimental design	Weak +	Improved children's vocabulary and parents' use of facilitative strategies

Neef (1997)	Very Low	+ Low risk of bias - Quasi-Experimental design - Inappropriately matched control group - Allocation Concealment/blinding - Incomplete outcome reporting - Undetermined validity of outcome measures	Weak +	Improved parent teaching skills and child skills development

Ozonoff & Cathcart (1998)	Very Low	+ Appropriately matched control group + Low risk of bias + Valid outcome measures - Quasi-Experimental design - lack of blinding - Incomplete outcome reporting	Weak +	Improved overall development, imitation, fine and gross motor skills and nonverbal conceptual skills

Rickards et al. (2000)	High	+ RCT + Appropriate randomization + Allocation Concealment/blinding + Clear outcome reporting + Low risk of bias + Valid outcome measures	Weak +	Improved children's IQ and behaviour problems

Schreibman et al. (1995)	Moderate	+ RCT + Appropriate randomization + Allocation Concealment/blinding + Low risk of bias + Valid outcome measures - Incomplete outcome reporting	Weak +	Improved parental affect while interacting with their child

Tonge et al. (2001)	High	+ RCT + Independent randomization + Allocation Concealment/blinding + Clear outcome reporting + Low risk of bias + Valid outcome measures	Weak +	Improved self-reported parent mental health

Wang (2008)	High	+ RCT + Appropriate randomization + Allocation Concealment/blinding + Clear outcome reporting + Low risk of bias + Valid outcome measures	Weak +	Improved the quality of parent's interactions with their child

* Recommendations are either Strong or Weak and For (+) or Against (-) the studied interventions; RCT = Randomized Controlled Trial

## Discussion

This pilot project provided evidence of the potential utility and feasibility of the streamlined GRADE process in the field of CYMH. Being the first implementation of this method in this area, it provides a unique contribution, though modifications to the process were required and certain limitations will have to be addressed in future studies in order for the GRADE method to achieve its potential in this field. A synthesis of the evidence relating to parent-implemented interventions for their child with autism was completed, along with an objective evaluation of this evidence. Following the evaluation of the evidence, recommendations were developed in detail to facilitate interpretation by decision makers for whom the recommendations are intended. This reporting should ensure that decision makers have all of the information required to judge the quality of a recommendation, determine its applicability, and adapt it, if necessary. Overall, it appeared that early, parent-implemented interventions for autism result in positive effects that outweigh any undesirable effects (though publication bias was not assessed and could have impacted the results of this study). However, as opposed to an overall recommendation for these interventions as a whole, specific recommendations had to be made based on the question of whether a particular intervention's benefits outweighed its harms, a necessary departure from the original GRADE method.

### Limitations

The first limitation may also be considered one of the strengths of this study. The GRADE approach was being implemented in a new field of research. Yet, as a result, there were no previous detailed applications or standards to draw from in the literature and modifications to the process were required. Some of the lessons learned from the current application of GRADE should inform future implementations of this approach in CYMH. This may also suggest that another panel may not reproduce the same results. In fact, others may disagree with aspects of our application to GRADE in this area of research and the subsequent grading of the quality of evidence and strength of recommendations. However, the transparency maintained by this process should allow others to understand the decisions made by this panel. For example, studies may be downgraded due to a lack of precision when there are very few patients or events, but there are no defined standards for these terms. Consequently, this panel followed the GRADE principle that a study may require greater precision in support of a recommendation when there is a close balance between the advantages and disadvantages of an intervention [66]. Given the lack of harmful events in the studied interventions, this was not the case here. Furthermore, the current panel consistently recommended the use of interventions for which there was no evidence of harm and some demonstrated benefit on one of the important outcomes. This is a particular judgment on the part of the current panel that has impacted the recommendations. Taken together, future iterations of this process may use panels with different values and judgments that would raise the threshold for demonstrated benefit as well as decide on a minimum sample size, which, if not met, would be downgraded for a lack of precision. Regardless of these decisions, they should continue to be made explicit and applied consistently.

The lack of variability in the composition of the panel may also be considered a limitation in the present application of GRADE. This panel was composed of clinical and methodological experts in the area of autism research. This may have reduced the variability of opinion in establishing the values and judgments and created a conflict of interest in reviewing and appraising evidence in their own field. This became a more prominent factor when the panel was required to appraise individual studies and provide individual recommendations for each intervention as part of the modified GRADE approach. However, these experts also had unique insight into the values and preferences of end-users, for which they were used as a proxy, as well as previous experience in this area of research that facilitated the timely completion of this pilot project. Moreover, these modifications to the process were necessary due to the evidence base and, again, all decisions were made transparent.

Examining the cause of the somewhat unsatisfactory finding, it is seemingly a product of the evidence and not due to any shortcomings in the GRADE method itself. The results of the studies could not be combined or compared due to the high degree of heterogeneity in the evidence base. Two interventions were rarely comparable in terms of the content of the intervention, outcomes measured, specific population studied or those implementing the interventions. Therefore, it was not always possible to construct a consistent account of the effects of an intervention. This is a common finding in mental health, and specifically autism research [33,36,45,48,49,67,68].

Research in this area has been identified as containing numerous gaps and being too heterogeneous to allow for direct comparisons of interventions [36,68]. A number of directions need to be pursued in order to allow for these comparisons. Specific areas to be pursued include: studying a wider range of outcomes; lengthening the time to follow-up assessments; assessing moderators and mediators of treatment outcomes; and, testing varied approaches to treatment delivery or even replicating past research [12,68,69]. An in-depth search of studies addressing these factors in relation to autism interventions was conducted for this pilot project and returned few results [e.g., [70,71]]. Studies addressing these factors would allow for mental healthcare providers and users to make the most informed decisions possible for each individual. Furthermore, the use of common, agreed upon outcome measures including child, parent, family and interactions variables, would facilitate the comparison of studies. Moreover, this research would produce more valuable information by using minimal standards in design and reporting on the interventions and participants. To this end, guidelines could be followed to improve the reporting of how studies were planned, conducted and analyzed [e.g., [72,73]].

The current approaches to synthesizing and evaluating this evidence cannot overcome the limitations in the research. An assumption of these approaches is that the development of the evidence-base has considered the fit between the intervention and its use in clinical practice. In reality, this fit has rarely been taken into account. Moreover, many make the assumption that all randomized control trials are of a high quality since they are generally considered the gold standard in research. The difficulty with this assumption is that there are situations where randomized control trials are impractical or unethical [31,74]. This is particularly true in some areas of CYMH research, where for a variety of logical reasons these designs are infrequently applied, such as autism [6]

In effect, the evidence-base in CYMH is primarily comprised of efficacy studies for which development is based on theory, methods and models to evaluate treatments that do not correspond to the demands of clinic and community-based care [75]. As a result, clinical research, and notably randomized controlled trials, does not provide practitioners with definitive answers for dealing with the individual-at-hand [76]. This may account for the discrepancy between the scientific support of interventions and the lack of their use in these settings [22,77-79].

### Strengths

The GRADE approach explicitly addresses the fit between the research and its use in practice through the functions of the panel, a clear strength. While research should still attend to the factors noted above, GRADE can focus on the clinical relevance of the considerable body of evidence already in existence. Another strength of this approach is that it does not reduce the evidence underlying an intervention to a simple Yes/No conclusion, but it includes information about key variables from the studies in the recommendations. This should aid in the interpretation of the strength of the recommendations and may affect clinical decision-making due to diverse values and preferences of individuals implementing the interventions. The short amount of time required to prepare the recommendations was also a strength of this process. It took four months to complete the process, whereas guideline development and systematic reviews can take up to two years or more [4,9,10].

This approach also benefits from an explicit methodology, transparency, consistency in judgments about the quality of evidence, and linking evidence to recommendations. Though evidence is linked to recommendations, the judgments about the quality of the evidence and the balance of benefits and harms leading to the recommendations are made separately. Not all grading systems separate decisions regarding the quality of the evidence from the strength of recommendations [19]. Those that fail to do so may create confusion. High quality evidence does not necessarily imply strong recommendations, and strong recommendations can arise from low quality evidence. GRADE also compels the panel to consider all relevant outcomes and to adhere to a consistent and comprehensive process in evaluating the evidence. This is a key advantage of this process, especially when considering that guideline developers have been found to lack objectivity due to excessive influence from industry and experts participating in the guideline panels, and to rarely abide by guidelines for the preparation of guidelines [8,80]

It should also be noted that GRADE has now been adopted by a number of organizations including the Cochrane Collaboration and the World Health Organization [28,53]. The increased usage of this methodology and presentation of its results, typically in summary of findings tables, may result in more rapid, clear and consistent translation of evidence. Furthermore, the results of one implementation of GRADE could be used in subsequent reviews quickly and transparently. For example, evidence from a GRADE review of interventions for other disorders on the autism spectrum could potentially be added to this review, while being downgraded for a lack of directness.

These strengths contribute to our experience that GRADE is a feasible approach in CYMH. Its methodology promotes reconciliation between clinicians', methodologists', administrators' and patients' perspectives along with the scientific evidence. Eliminating this traditional separation, seen as a significant limitation in clinical practice guidelines and systematic reviews, will help the evolution of the CYMH field toward the use of evidence-based practice by service providers.

### Future Directions

Future implementations of the GRADE approach should include a broader representation of stakeholders in the panel than was used in this pilot project. Other members of the panel could include those of varying levels of service administration such as policy makers, parents, and other practitioners. They would provide more varied perspectives and reduce the gap between the evidence and practice. For example, considering practical barriers to implementation, such as funding restrictions or gaps in clinical expertise could be especially helpful in making the most useful recommendations. In addition, including stakeholders of varying levels would help to drive the knowledge exchange of the findings. Knowledge exchange is an area in and of itself that should be explored to provide guidance on the best procedure for translating results in order to produce meaningful change when using the GRADE method [81]. Other considerations in future uses of GRADE include having the details of the manualization of the intervention as part of the evaluation of methodological quality. Manuals serve the important objective of making the recommended treatments available for clinical training and practice. The specification of sample characteristics in the study could also be evaluated in the GRADE process. These details reflect the recognition that specific treatments may be efficacious only within a limited range of individuals depending on such factors as their age, sex, problem severity, socioeconomic status, and ethnicity [82].

## Conclusions

In conclusion, it was determined that it is feasible to develop more timely recommendations in CYMH, specifically early parent-implemented autism interventions, using a modified version of the systematic and transparent GRADE approach. A definitive answer as to which is the best intervention was not produced. However, the GRADE approach appeared capable of providing a specific answer had the evidence been less heterogeneous and directly comparable. Due to the heterogeneity of the evidence a modified GRADE approach was presently evaluated. As research in this area increases and fills in the gaps identified above, it is important to have a method to evaluate the benefits and risks of new interventions, in an efficient manner, as they arise. The GRADE process meets this need and establishes credible knowledge that leads directly to recommendations for effective organization and delivery of CYMH care. Most importantly, GRADE and the recommendations developed from it, will help CYMH service providers and users to make treatment decisions that are informed by the scientific and practice-based evidence.

## Competing interests

The authors declare that they have no competing interests.

## Authors' contributions

JMVA conceived of the study, and participated in its design and coordination and drafted the manuscript. JDG, AP, and JAMR were expert panel members and as such contributed significantly to the findings and also contributed to the study design, and edited the manuscript. All authors read and approved the final manuscript.

## Pre-publication history

The pre-publication history for this paper can be accessed here:

http://www.biomedcentral.com/1472-6963/11/60/prepub

## Supplementary Material

Additional file 1 **Appendix - Sample Recommendation**. Example of recommendation from the pilot-project Click here for file
