# Supplementary material for: Credible knowledge: A pilot evaluation of a modified GRADE method using parent-implemented interventions for children with autism
Source: BMC Health Serv Res. 2011 Mar 22;11:60. doi: 10.1186/1472-6963-11-60 (PMC3072313; doi:10.1186/1472-6963-11-60)
Supplement: Additional file 1 — Appendix - Sample Recommendation. Example of recommendation from the pilot-project [file 1472-6963-11-60-S1.DOC]

APPENDIX

Question: Should a social communication parent training intervention along with routine care be used for autistic children?

Comparison Group: Autistic children receiving routine care alone.

Setting: U.K. Instructed in the clinic and administered in the home.

Reference: Aldred, C., Green, J. & Adams, C. (2004) A new social communication intervention for children with autism: a pilot randomised controlled treatment study suggesting effectiveness. Journal of Child Psychology and Psychiatry, 45, 1420–1430.

Outcomes: Various parent-child interaction measures, observation measure of child communication, parent report measures of communicative development and adaptive behaviour, and a standardized diagnostic measure representing symptom severity.

Evidence Summary: Randomized controlled trial evaluating a theoretically based social communication intervention that targeted parental communication compared to routine care alone. This manualized intervention was given in addition to existing care and utilized psycho-education workshops with the parent and child for six sessions over six months with a further six months of 2-monthly consolidation sessions. The aim of the intervention is to educate parents and train them in adapting their communication style to fit the individual competencies of their child. Fidelity of implementation was evaluated through attendance records and asking parents to spend 30 minutes per day working with their child. Evaluations of comorbidity and language level of the child were not performed. Children were aged 2 years to 5 years 11 months (n = 28). Outcomes were measured at 12 months from the start of the intervention.

Quality of Evidence: This was a HIGH quality randomized control trial. It was noted that parents had an above average level of education but this was not deemed sufficient to downgrade the evidence.

Best Estimates: Improved symptom severity, social communication skills and parent’s perceptions of their child’s communicative skills in the intervention group compared to the control group.

Grade of Recommendation: For parents with autistic children in the age range studied we suggest the use of the social communication intervention described in this study in conjunction with existing care for improving symptom severity, social communication skills and parent’s perceptions of their child’s communicative skills (weak recommendation based on high quality evidence). This weak recommendation applies in comparison to a control group receiving routine care alone. It should be noted that a critical child outcome (standardized direct testing measure of IQ) was not evaluated in this study. Future research should seek to evaluate this critical outcome and include a larger independent sample so that more comprehensive recommendations can be made.
